# Mechanical stress induces elastic fibre disruption and cartilage matrix increase in ligamentum flavum

**DOI:** 10.1038/s41598-017-13360-w

**Published:** 2017-10-12

**Authors:** Kazunori Hayashi, Akinobu Suzuki, Sayed Abdullah Ahmadi, Hidetomi Terai, Kentaro Yamada, Masatoshi Hoshino, Hiromitsu Toyoda, Shinji Takahashi, Koji Tamai, Shoichiro Ohyama, Akgar Javid, Mohammad Suhrab Rahmani, Maruf Mohammad Hasib, Hiroaki Nakamura

**Affiliations:** Department of Orthopaedic Surgery, Osaka City University Graduate School of Medicine, Osaka, Japan

## Abstract

Lumbar spinal stenosis (LSS) is one of the most frequent causes of low back pain and gait disturbance in the elderly. Ligamentum flavum (LF) hypertrophy is the main pathomechanism of LSS, but the reason for its occurrence is not clearly elucidated. In this study, we established a novel animal model of intervertebral mechanical stress concentration and investigated the biological property of the LF. The LF with mechanical stress concentration showed degeneration with elastic fibres disruption and cartilage matrix increase, which are similar to the findings in hypertrophied LF from patients with LSS. By contrast, decreased *Col2a1* expression was found in the LF at fixed levels, in which mechanical stress was strongly reduced. These findings indicate that mechanical stress plays a crucial role in LF hypertrophy through cartilage matrix increase. The findings also suggest that fusion surgery, which eliminates intervertebral instability, may change the property of the LF and lead to the relief of patients’ symptoms.

## Introduction

Lumbar spinal stenosis (LSS) is one of the most frequent causes of low back pain, and gait disturbance seriously affects the activities of daily living and health in the elderly population. In patients with LSS, dural sac, cauda equina, or nerve-roots are compressed by hypertrophied ligamentum flavum (LF) and protruded lumbar discs, which mainly lead to back and leg symptoms^1–3^.

Several studies have investigated the degeneration of the LF in patients with LSS using the tissue harvested during spine surgery; however, the molecular mechanism of LF hypertrophy has not been determined yet^4–9^. One of the major problems of those studies was the uncertainty of factors that may be related to LF hypertrophy, such as age, sex, disease severity, disease duration, and comorbidities of patients. To elucidate the pathomechanism of LF hypertrophy, investigation should be performed under standardised conditions.

In clinical situations, instability of affected motion segment is frequently observed in patients with LSS, and this motion increase is speculated to have a crucial relationship with the pathogenesis of LSS^9–12^. Hur *et al*.^9^ reported that segmental range of motion in the lumbar spine has a weak but significant correlation with LF thickness in patients with LSS. Yoshikawa *et al*.^10^ also showed that increasing segmental range of motion is one of the independent risk factors of LF thickening.

In this study, we aimed to establish a novel animal model for degeneration and hypertrophy of the LF using mechanical stress concentration. We also aimed to investigate the effect of mechanical stress on LF degeneration to elucidate the pathomechanism of LF hypertrophy.

## Materials and Methods

### Data availability statement

All data used for this study are publicly available and accessible online.

### Experimental design

All surgical and experimental procedures were approved by the ethics committee of Osaka City University Graduate School of Medicine, and performed in accordance with the guidelines for Laboratory Animal Center (approval number: 15007).

Eighteen-week old male New Zealand White Rabbits (2800–3200 g) were randomly divided into three groups. The first group underwent L2-3 and L4-5 posterolateral fusion with instrumentation (PLF; the details are in the next section) to obtain mechanical stress concentration in L3-4 level (group F; n = 10). The second group underwent additional resection of L3-4 supraspinal muscle after the same PLF procedure of group F (group F + C; n = 6). The L3-4 level in group F + C was expected to concentrate more intervertebral mechanical stress than that in group F following operation. The third group just underwent surgical exposure as a sham operation (group S; n = 6). All rabbits were housed individually with free access to food and water. Three groups of rabbits were sacrificed at 16 weeks after surgery. In addition, three more rabbits without operation were sacrificed, and the specimens were prepared for histological analysis as another control (pre-treatment group).

### Surgical procedure

**Figure 1:**
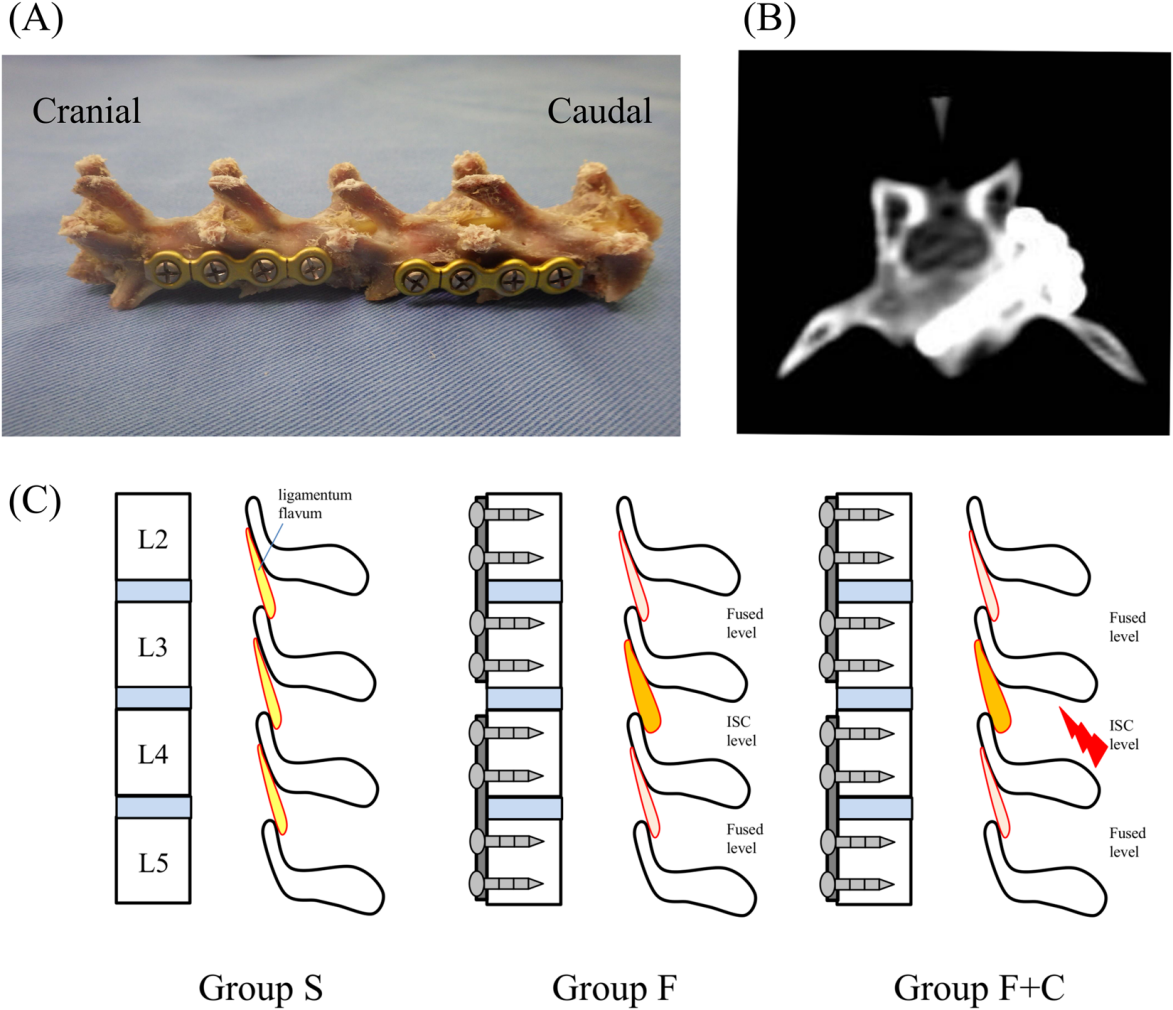
Implant and procedure of PLF: (**A**) Left view of L2-3 and L4-5 fused spine, (**B**) Postoperative axial CT image of L3 inferior screw, (**C**) Schema of three experimental groups.

All surgical intervention was performed with sterile instrumentation using aseptic techniques. After anaesthesia (an intramuscular injection of 30 mg/kg of ketamine hydrochloride and 10 mg/kg of xylazine) and injection of antibiotics (20 mg/kg of Flomoxef Sodium; Shionogi, Osaka, Japan), a dorsal midline skin incision was performed following X-ray control. Lumbosacral fascia was cut just lateral to the mammillary process, and only left-sided intermuscular plane between the multifidus and longissimus muscles was developed. Subsequently, the multifidus muscle was detached from lateral to the mammillary processes, while the lamina and base of the transverse process, posterior edge of vertebral body, were exposed. In group S, fascia, subcutaneous tissue, and skin were then closed using 4-0 polydioxanone absorbable suture and 3-0 non-absorbable nylon suture following careful haemostasis and irrigation with saline solution. In groups F and F + C, each 4-hole titanium locking plate (Universal Mandibular System; Leibinger, Stuttgart, Germany) was placed on the posterolateral side of L2-3 and L4-5, and a 2.0-mm titanium locking screw was then inserted into each vertebra and locked as described in our previous study^13^ (Fig. 1A–C). Decortication was not performed, and all fixation procedures could be completed without direct visualization or touching of the LF to minimise tissue damage. In group F + C, additional L3-4 supraspinal muscle resection was undergone after PLF. In a rectangular scope (2 cm width, 1 cm depth), supraspinal muscle was sharply dissected. Wound closure in groups F and F + C was performed following the same procedure as for group S.

### Intervertebral range of motion

**Figure 2:**
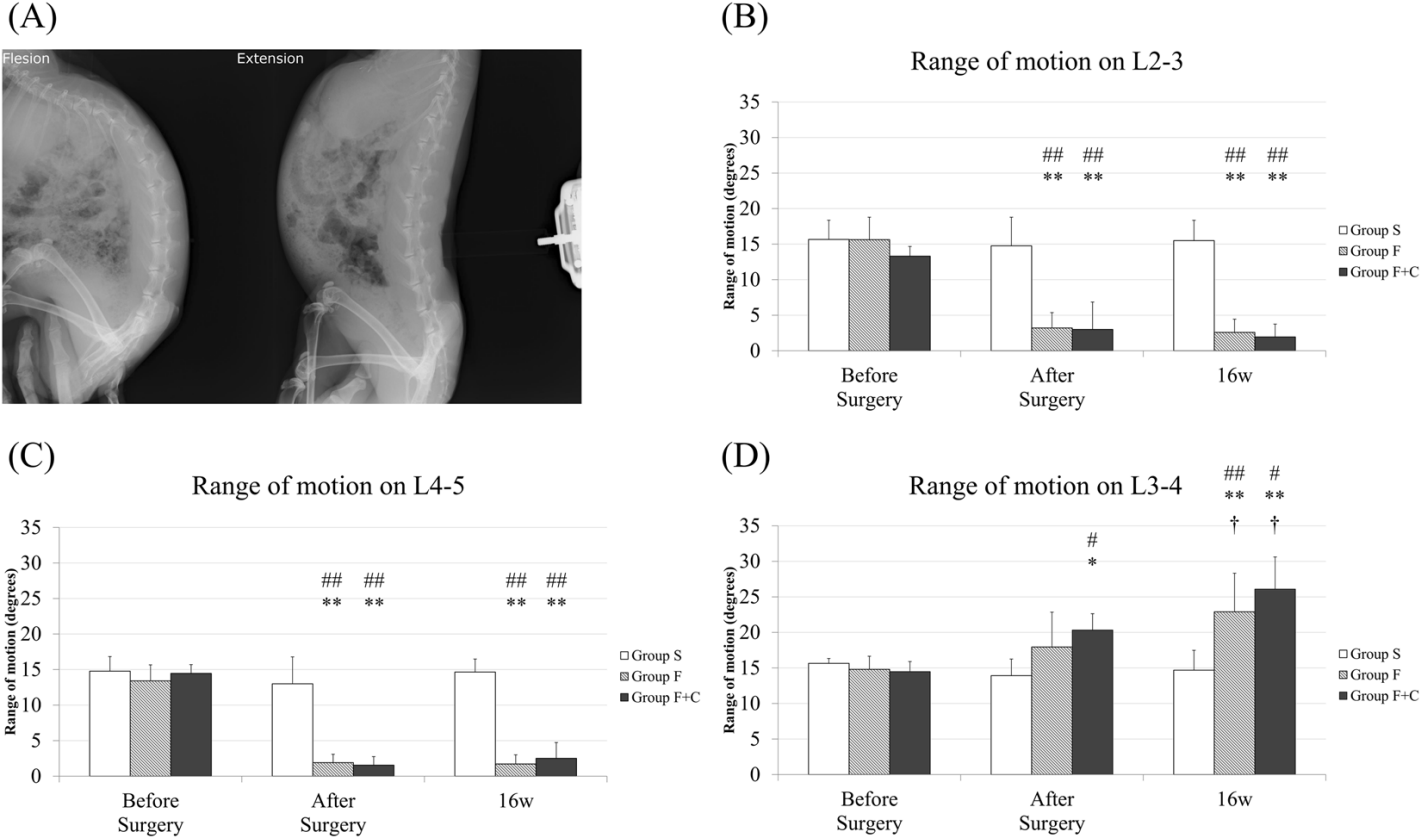
Evaluation of the range of motion with dynamic X-rays. (**A**) Representative plain X-ray in flexion and extension position, (**B**) L2-3, (**C**) L4-5 and (**D**) L3-4 range of motion. (*p < 0.05 and **p < 0.01: compared with before surgery in the same group; ^†^p < 0.05: compared with after surgery in the same group; ^#^p < 0.05 and ^##^p < 0.01: compared with group S in the same time points).

Intervertebral range of motion from L2-3 to L4-5 was evaluated by lateral view of manipulated dynamic X-ray under anaesthesia. To make the condition constant, in flexion position, pressure due to a force of 10 N was placed on T9 spinal process using a pressure measuring device (Imada Co., Aichi, Japan), while the iliac bone was held by hand. In the extension position, pressure due to a force of 20 N was placed on L3 spinal process while the chest and iliac bone was held (Fig. 2A). Each X-ray view was taken before and immediately after surgery, and 16 weeks in all the cases. The data were sent to a picture archiving communication system (PACS), and intervertebral motion was measured between the lines along the lower endplate of each vertebra using ImageJ 1.50 (public domain).

### Histological and immunohistological evaluation

Spinal motion segments with the left side on the LF from L2-3 to L4-5 were harvested and fixed with 4% paraformaldehyde phosphate buffer solution. Subsequently, the specimen was decalcified in Morse solution (Wako, Osaka, Japan) for three weeks, dehydrated, and embedded in paraffin. Sections from L2-3 to L4-5 segments with the facets and LF were sliced along the coronal plane at the junction of the right and left inferior articular processes to standardise the sliced section in each specimen (4 µm thick). After dewaxing, the sections were stained with haematoxylin and eosin (HE), elastica van Gieson (EVG), and toluidine blue (TB). The sections were examined with a Digital Image Analyzer (Olympus, Tokyo, Japan). In further analysis, we compared the LF obtained on the L3-4 level of groups F, F + C (intervertebral stress concentration level; ISC level), and S. We also compared the LF obtained on L2-3 and L4-5 levels in groups F, F + C (fused levels), and S (Fig. 1C). The thickness of the LF was evaluated by measuring the area of the LF using HE staining to trace the edge of the LF. The elastic and collagen fibres stained black and red, respectively, in the EVG stain. The density of the elastic fibres in the LF was calculated by the percentage of area that stained black relative to the whole area of the LF with EVG stain. Cartilage matrix increase on the LF was analysed by the area stained purple on the LF with TB stain, while the cartilage in the facet joint was considered as a control. The area of LF and corresponding stained regions were measured using Image J 1.50.

For collagen type II alpha 1 chain (Col2a1) immunostaining, sections were retrieved with 10% antigen retrieval solution (Dako, Carpentaria, CA) and 2% hyaluronidase (Wako, Osaka, Japan). Endogenous peroxidase activity was quenched in 30% H_2_O_2_ for 30 min, and endogenous immunoglobulin was then blocked with 10% goat serum (Nichirei, Tokyo, Japan). After incubation with Col2a1 mouse monoclonal antibody (1:50; Kyowa Pharma Chemical, Takaoka, Japan) for 1 h, secondary antibody (1:100; Vector Laboratories, Burlingame, CA) incubation was carried out for 2 h. The reaction was visualised using the Vectastain ABC kit (Vector Laboratories, Burlingame, CA) and diamino benzidine (WAKO, Osaka, Japan). Sections were counterstained using haematoxylin. Col2a1 immunoreactive cells were counted in the whole area of the LF in each specimen, and the cartilage of the facet joint was regarded as a positive control. Negative control was the cartilage of the facet joint in the specimen prepared with same procedure without primary antibody.

### Disc height measurement

The height of the L3-4 disc was evaluated using computed tomography (CT) with the rabbits in a prone position and under anaesthesia. CT imaging was performed on a single slice helical scanner (Prospeed AI; GE Healthcare, United Kingdom); slice thickness was 1 mm. Each scan was taken immediately and 16 weeks after surgery. Of 22 rabbits, 1 from group S and 3 from group F were excluded because of the poor quality of images. The data were sent to PACS and reconstructed in the sagittal plane vertical to the disc. Anterior, middle, and posterior straight lines of the discs were drawn, and the distances on the PACS measured. Each disc height was calculated using the average of the three distances (see Supplementary Fig. 1A). We also evaluated the relationship with scatter plots between the disc height loss between immediately and 16 weeks after surgery, and histological findings of the LF in each specimen.

### Quantitative real-time polymerase chain reaction (PCR)

Immediately after the rabbits were sacrificed, the deep layers in the central portion of the right side on the LF from L2-3 to L4-5 were immersed in RNA-storing reagent (RNAlater solution; Ambion, Austin, TX) and stored at -20 °C until subsequent experiments were performed^14^. The total RNA from LF tissues was isolated using QIAzol Lysis Reagent and RNeasy Plus Universal Mini Kit (Qiagen, Venlo, Netherlands) according to the manufacturers’ protocol. After the concentration and purity were measured, cDNA was synthesised using a synthesis kit (iScript, Bio-Rad, Hercules, CA).

To determine RNA levels in the LF samples of each lumbar level in the three experimental groups, quantitative real-time PCR kits (SYBR Premix Ex Taq, Takara, Shiga, Japan) were used. For PCR, the reaction mix contains 10 μL of SYBR premix EX Taq2 with 0.8 μL of forward and reverse primers, 0.4 μL of ROX Reference Dye2, 8 μL of diluted reverse transcription products, and nuclease-free water. All reactions were performed in triplicates on a 7500 Fast Real-time PCR system (Applied Biosystems, Foster City, CA) with the following conditions: 95 °C for 30 s, followed by 40 cycles at 95 °C for 3 s, and 60 °C for 1 min. β actin was used as an endogenous control after preparation of three other prospective endogenous genes in LF tissue (GAPDH, 18 s, HPRT-1, data not shown). The threshold cycle (Ct) value is defined as the cycle number at which the fluorescence exceeds the given threshold, and comparative Ct was used for evaluation of gene expression. We evaluated the expression of genes related to cartilage (*Col2a1*), elastic fibres (*Elastin*), and fibrosis (*Col1a1*, *Col1a2*, and *Col3a1*). The primers are listed in supplemental Table 1.

### Statistical analysis

All statistical analyses were performed with EZR (Saitama Medical Center, Jichi Medical University, Saitama, Japan), which is a graphical user interface for R (The R Foundation for Statistical Computing, Vienna, Austria; version 2.13.0). Repeated ANOVA with Bonferroni correction was used to access intervertebral range of motion and disc height measurement in the same specimen. One-way ANOVA with Bonferroni correction was used to assess RT-PCR analysis. Kruskal-Wallis analysis with multiple consumption of Steel-Dwass was performed to assess the histological analysis in each specimen (significance at p < 0.05).

## Results

### Mechanical stress concentrates on L3-4 with increasing range of motion in groups F and F + C

The range of motion of L2-3, L3-4, and L4-5 in each group is shown in Fig. 2B–D. In L2-3 (Fig. 2B) and L4-5 (Fig. 2C) of groups F and F + C, the range of motion was almost disappeared immediately after surgery and maintained less than 5 degrees at 16 weeks (fused levels). By contrast, in L2-3 and L4-5 of group S, the range of motion was unchanged during the study periods. On the other hand, in L3-4 of group F + C, the range of motion was significantly increased immediately after surgery compared with that at before surgery (p = 0.03). Further increase was also observed over 16 weeks compared with that immediately after surgery (p = 0.03, ISC level; Fig. 2D). In L3-4 of group F, while the range of motion at 16 weeks showed significant increase compared with that at before surgery (p = 0.001), the difference in the range of motion between before surgery and immediately after surgery did not reach significance (ISC level). Instead, the range of motion in L3-4 of group S was unchanged during the study periods. In comparison of the range of motion in L3-4 between the groups, group F + C was significantly larger than group S at immediately after surgery (p = 0.01), and both groups F and F + C were significantly larger than group S at 16 weeks after surgery (p = 0.005, 0.01, respectively).

### Mechanical stress concentration induces the thickening of the LF

**Figure 3:**
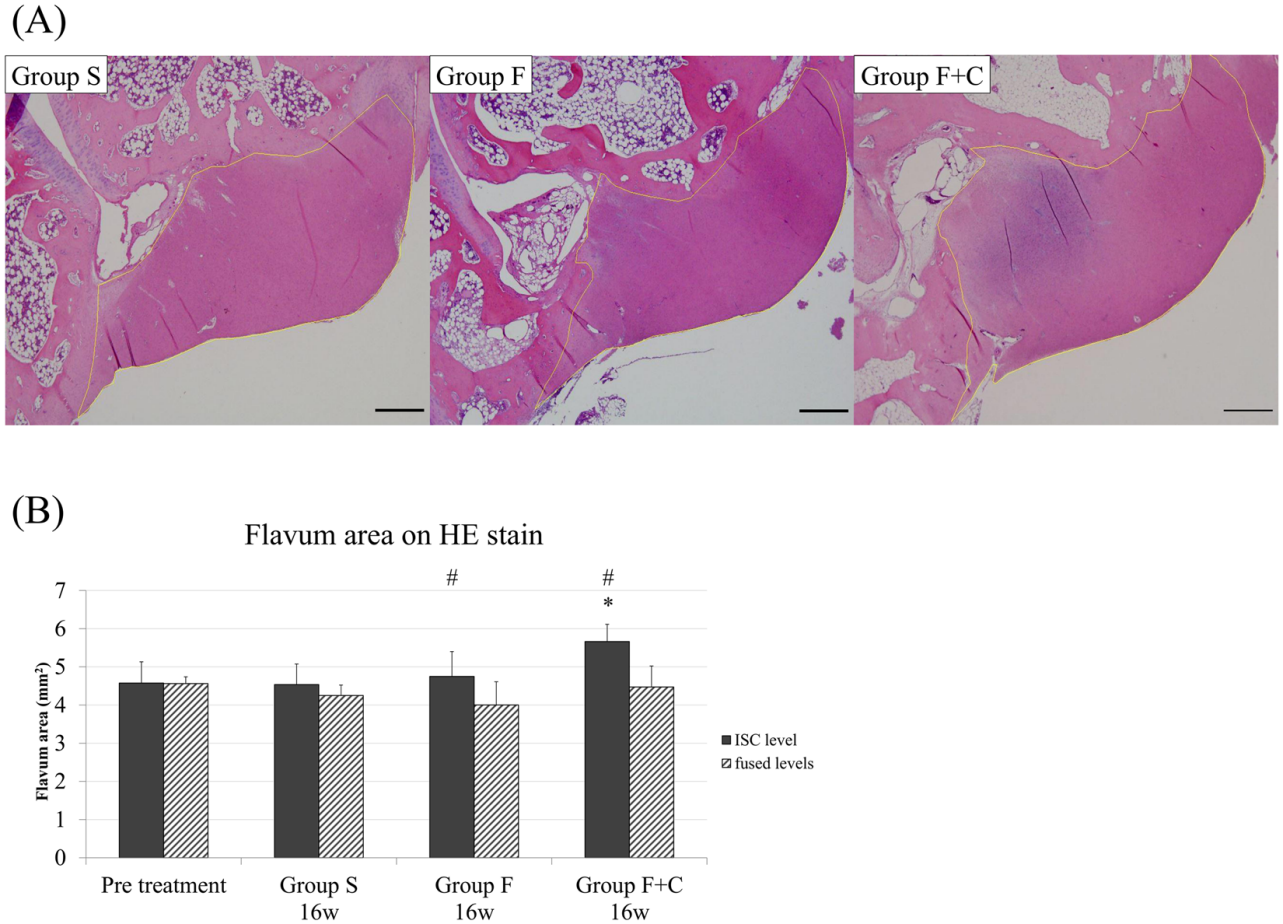
HE staining: (**A**) L3-4 LF in each group (×20). Orange line indicates the border of the LF, (**B**) Cross sectional area of the LF. (*p < 0.05: compared with the other levels in the same group; ^#^p < 0.05: compared with the same level of group S).

The thickness of the LF was evaluated to compare the area of the LF in each specimen with HE stain (Fig. 3A). The ISC level LF in groups F and F + C was significantly thicker than the fused levels in the same groups (p = 0.02 and 0.01, respectively), and the ISC level LF in group F + C but not in group F was significantly larger than that on the same level in group S at 16 weeks (p = 0.04; Fig. 3B). In contrast, we could not observe any shrinkage of the LF on fused levels in group F or F + C compared with the same level in group S.

### Histological evaluation reveals elastic fibres disruption and cartilage matrix increase in LF by mechanical stress

**Figure 4:**
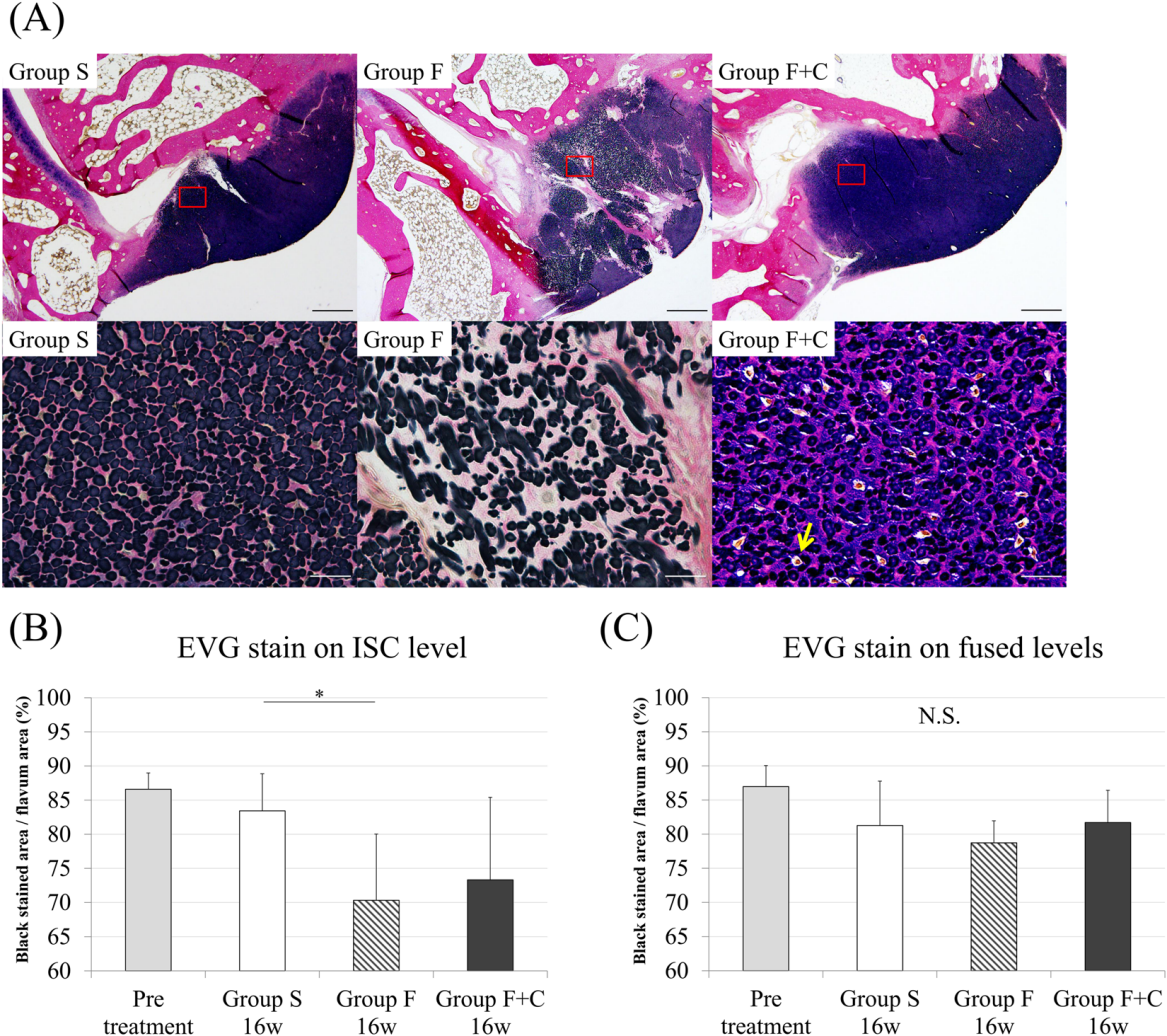
EVG staining. (**A**) L3-4 LF in each group (upper row: ×20, lower row: ×400), (**B**) Density of elastic fibres in L3-4 LF. (**C**) Density of elastic fibres in L2-3 and L4-5 LF. (*p < 0.05: compared with same level in group S; N.S: no significant difference).

With EVG stain, the density of elastic fibres on the LF was evaluated. The ISC level LF at 16 weeks in group F showed significant decrease in the density of elastic fibres compared with the same level in group S (p = 0.03), but the difference between groups F + C and S did not reach statistical significance (Fig. 4A, B). However, on the area around the facet joint, increases in collagen and chondrocyte-like cells were frequently observed on the ISC level LF in group F + C but not on the same level in group S (Fig. 4A). On fused levels, the LF in group F or F + C did not show a decrease or an increase of elastic fibres compared with the same levels in group S (Fig. 4C). Meanwhile, on the area around the facet joint, the collagen fibre was prominently obscure compared with that of group S.

**Figure 5:**
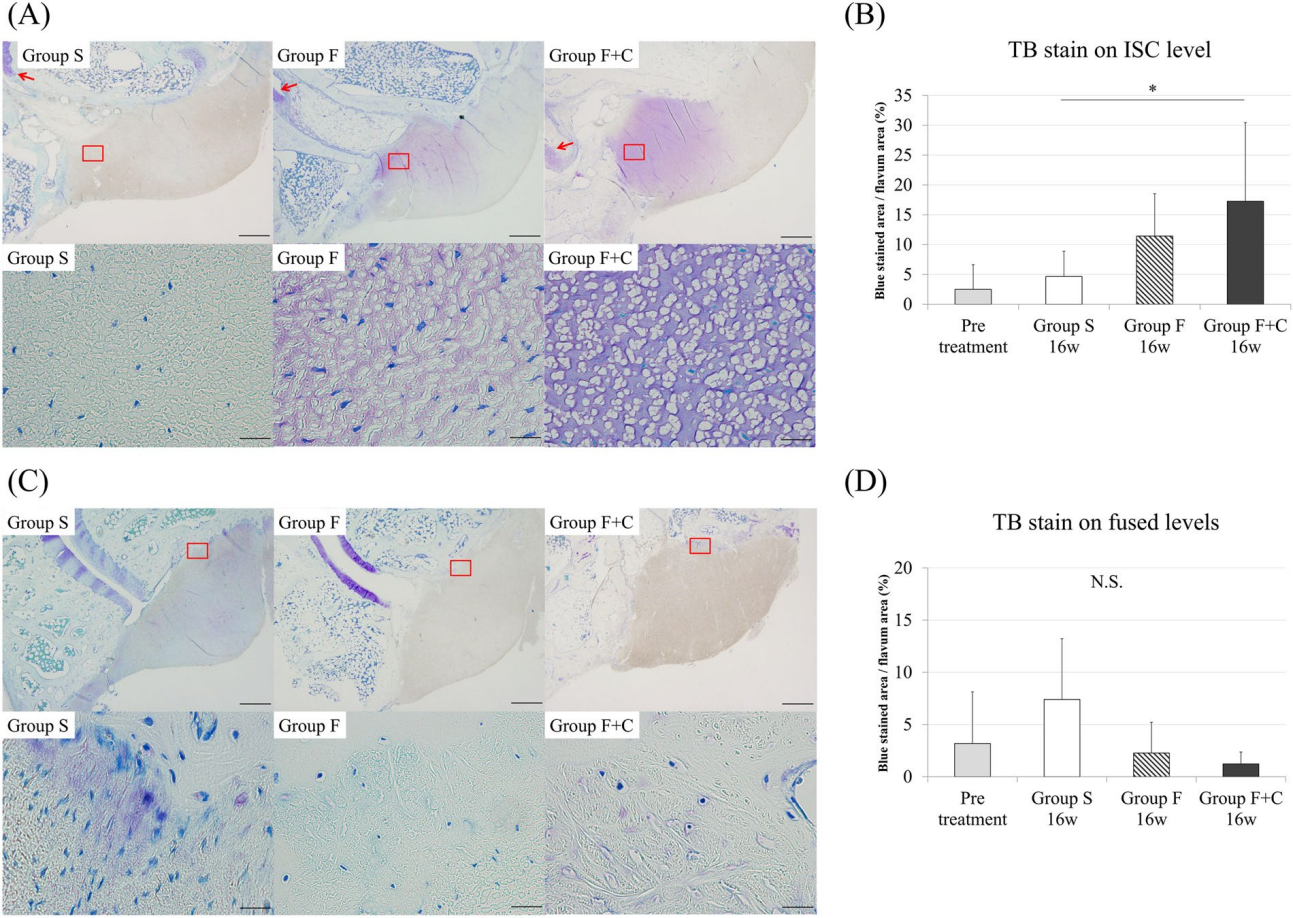
TB staining. (**A**) L3-4 LF in each group (upper row: ×20, lower row: ×400). Red arrows indicate facet joint cartilage. (**B**) Cartilage matrix area in L3-4 LF. (**C**) L2-3 LF in each group (upper row: ×20, lower row: ×400), (**D**) cartilage matrix area in L2-3 and L4-5 LF. (*p < 0.05: compared with same level in group S; N.S: no significant difference).

For the cartilage matrix increase on the LF, evaluated with TB stain, the area containing the cartilage matrix on the ISC level LF in group F + C was significantly larger than that on the same level in group S (p = 0.04; Fig. 5A, B). The ISC level LF in group F + C showed purple staining, which indicates cartilage matrix from the edge to the centre of the LF around the facet joint. By contrast, we could not observe the purple-stained area of the LF in group S, except the insertion from the facet joint. The area on the ISC level LF in group F + C stained purple was almost same in the area where collagen increase was obtained on EVG staining. On fused levels, although no significant reduction of cartilage matrix was found in group F or F + C compared with that in group S, the purple-stained area tended to disappear at the insertion of the LF from the facet joint (Fig. 5C,D).

### Col2a1 immunoreactive cells increase identified on the ISC level in group F + C

**Figure 6:**
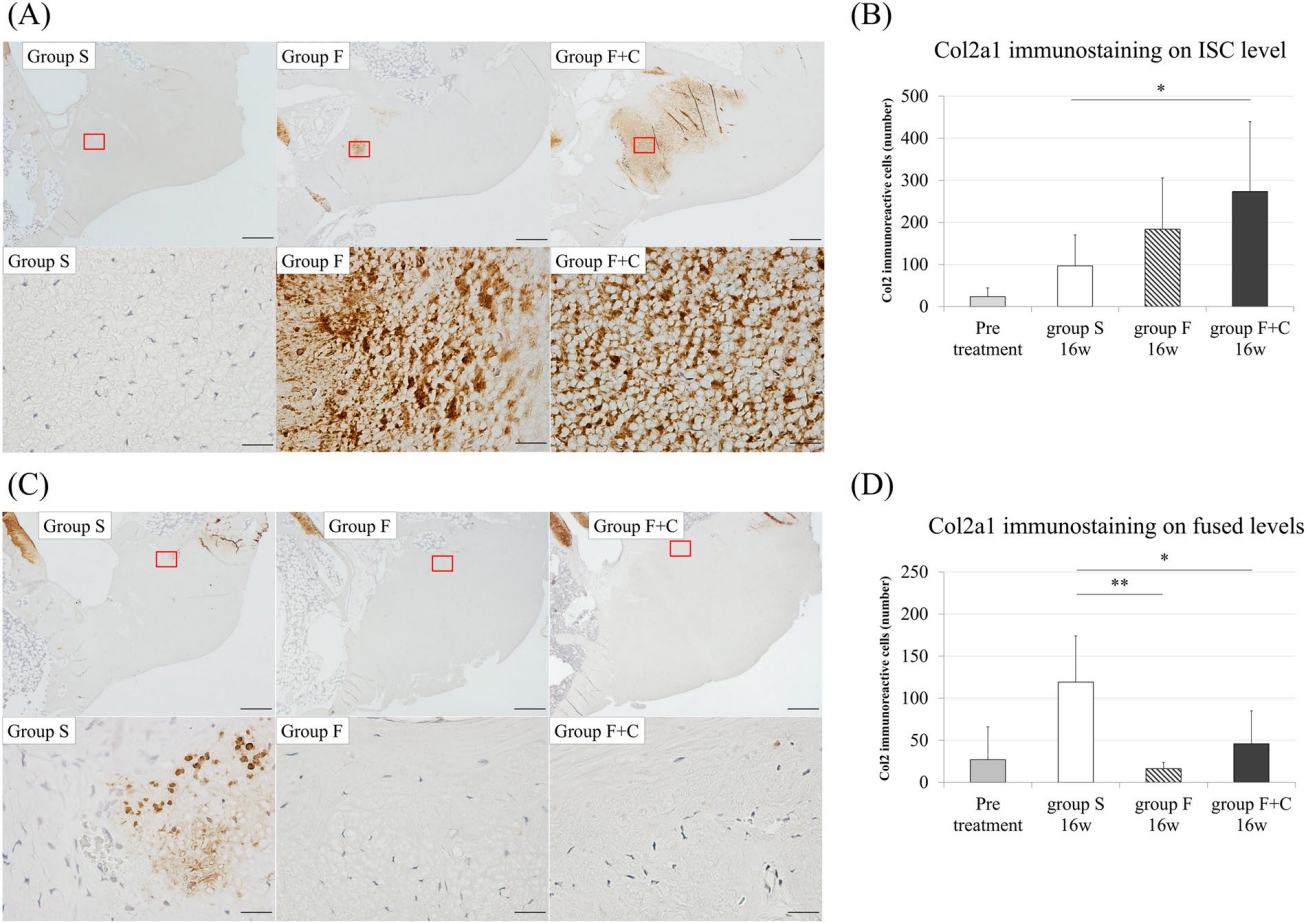
Col2a1 immunohistological stain (**A**) L3-4 LF in each group (upper row: ×20, lower row: ×400). (**B**) Number of immuno-positive cells in L3-4 LF, (**C**) L2-3 LF in each group (upper row: ×20, lower row: ×400), (**D**) Number of immuno-positive cells in L2-3 and L4-5 LF. (*p < 0.05: compared with same level in group S; N.S: no significant difference).

Representative specimens of Col2a1 immunohistological staining and immunoreactive cell counts are shown in Fig. 6. The ISC level LF in group F + C had significantly larger number of Col2a1 immunoreactive cells than that on the same level in group S (p = 0.04; Fig. 6A,B). In addition, the fused levels LF in groups F and F + C had significantly smaller number of immunoreactive cells than that on same level in group S (Fig. 6C,D).

### Disc height change and the relationship with LF degeneration

Disc height decreased at 16 weeks, compared with that immediately after surgery in groups F and F + C, whereas no significant difference was noted that in group S (see Supplemental Fig. 1b–d). Scatter plots demonstrated that disc height loss had a weak but significant linear relationship with the number of *Col2a1* immunoreactive cells (Supplemental Fig. 1e).

### *Col1a2* and *Col2a1* gene expression increases with mechanical stress concentration on LF

**Figure 7:**
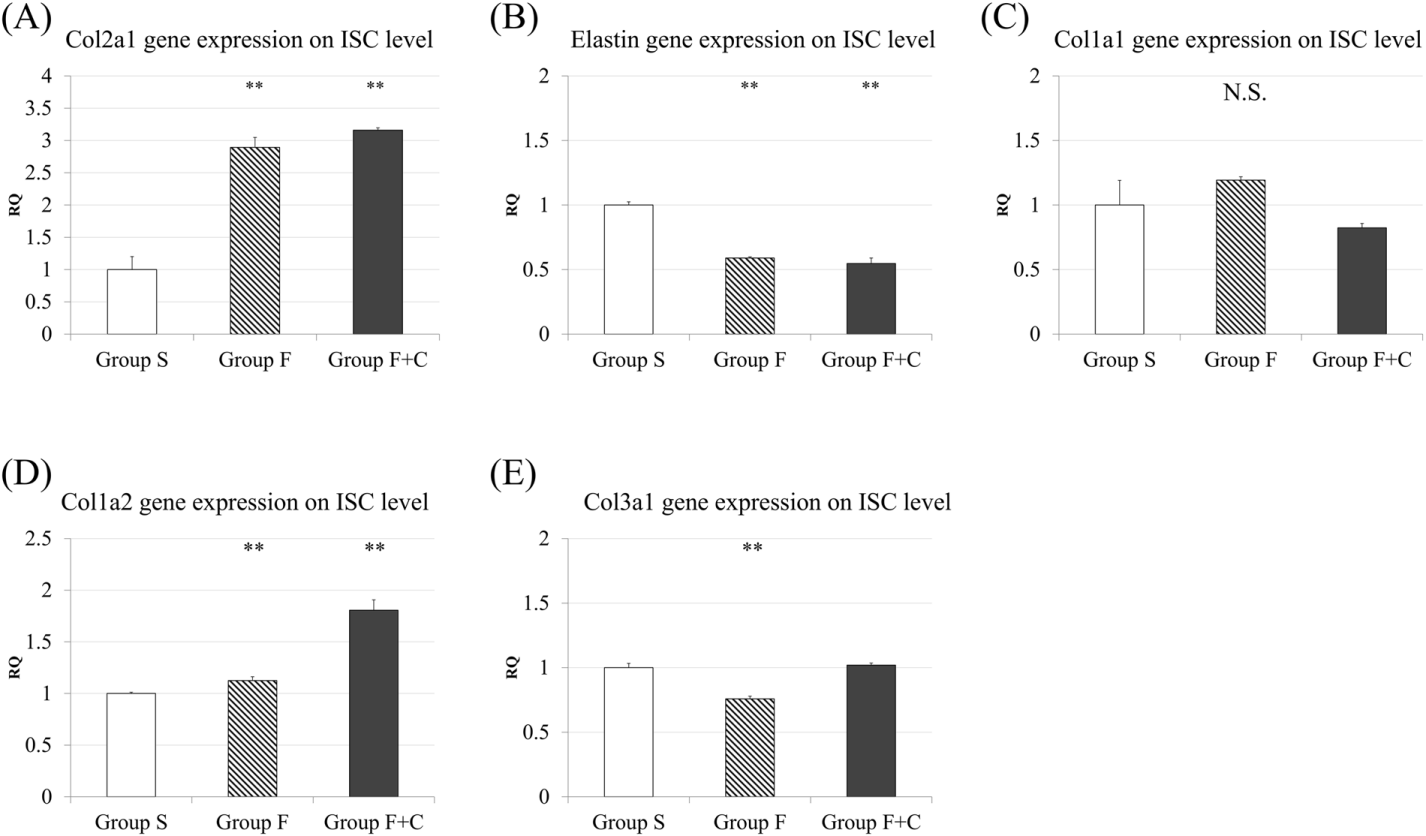
mRNA expression profile of the LF: (**A**) *Col2a1* (**B**) *Elastin* (**C**) *Col1a1* (**D**) *Col1a2* (**E**) *Col3a1* expression in L3-4 LF. (*p < 0.05 and **p < 0.01: compared with group S; N.S.: no significant difference; RQ indicates relative quantity).

The levels of mRNA expression in each group were evaluated by quantitative real-time PCR. On the ISC level LF in group F or F + C, the *Col2a1* expression was approximately threefold increments compared with that on the same level in group S, while *Elastin* expression was significantly decreased (p < 0.01) (Fig. 7A,B). In addition, the *Col1a2* expression on the ISC level in group F + C showed significant increments compared with that on the same level in group S (p < 0.01); no apparent change was observed in *Col1a1* or *Col3a1* (Fig. 7C–E). On the other hand, both *Col2a1* and *Elastin* levels were significantly lower on the fused levels LF in groups F and F + C than those on the same level in group S (p < 0.01) (Supplemental Fig. 2A,B). Almost all of the fibrosis-related collagen gene expression was downregulated on the fused level in group F or F + C, compared with the same level in group S (Supplemental Fig. 2C–E).

## Discussion

In this study, we established a rabbit model in which mechanical stress was concentrated on L3-4 segment with adjacent segment fusion (L2-3 and L4-5), and evaluated the change in the biological property of the LF. We also prepared an additional L3-4 supraspinal muscle resection model, which was expected to concentrate much more stress on L3-4. With these rabbit models, we were able to assess the effect of the increase and decrease of mechanical stress on the LF. To our knowledge, no experimental investigation that could clarify the effect of mechanical stress on the LF has been carried out.

Manipulative dynamic X-ray showed that a range of motion increase was observed over 16 weeks on the ISC level, whereas a significant decrease was achieved on fused levels both in groups F and F + C. In addition, earlier motion increase was obtained after surgery on the ISC level in group F + C compared with group F to cut the supraspinal muscle additionally. These results indicate that an ideal experimental intervertebral stress concentration was obtained using these fixation models on the ISC level, and experimental intervertebral stress shielding was also obtained on fused levels.

In earlier studies, the LF obtained from patients with LSS who underwent spine surgery has been reported to express fibrosis or chondrogenesis with the change in the density of elastic fibres^4–7^. Sairyo *et al*.^5^ reported fibrosis in the entire area of the LF in patients with LSS. They advocated that inflammatory scarring could not explain the entire mechanism but might be one of the mechanisms of LF hypertrophy. In contrast, Yoshida *et al*.^6^ compared the LF from patients with LSS and that from patients with lumbar disc herniation (LDH) as a control. They initially reported proliferation of chondrocytes in 91% of LSS tissue with the increase ofCol2. Yabe *et al*.^7^ performed cDNA microarray analysis of LSS tissue samples and concluded gene expression related to chondrogenesis. They found that proteoglycan synthesis was higher in the LF from patients with LSS, whereas no difference in the gene expression related to inflammation was found. Our previous report demonstrated that the cell number and cartilage matrix area were significantly higher in hypertrophied LF from patients with LSS compared with those from patients with LDH; these changes were more evident in the tissue from patients with LSS with segmental instability^4^. Aside from these theories, some authors proposed that angiogenesis mediated from angiopoietin-like protein-2 or vascular endothelial growth factor has a crucial role for LF hypertrophy^8,9^.

Compared with previous studies using human specimens, we would be able to obtain more accurate data regarding the biological reaction of the LF to mechanical stress using a rabbit model under standardised condition. The normal LF in humans is a well-defined elastic structure with 80% elastic fibres and 20% collagen fibres^15^. The LF in the lumbar region of rabbits was also reported to obtain thick elastic fibres arranged in parallel^16^. In this study, the density of elastic fibres with EVG stain in group S and pre-treatment group was almost equal to that of the normal LF in humans. Based on these findings, the structure of the LF in rabbits is similar to that in humans and using a rabbit model for the study of the LF is considered reasonable.

In the present study, we performed a comparison on LF of three groups in 16 weeks after surgery. We found that the ISC level LF in group F + C was significantly thicker than that in group S. This result suggests that the LF would be degenerated and hypertrophied under the continuous increase of mechanical stress like this model. With TB stain, significant cartilage matrix increase was observed on the ISC level in group F + C compared with that on the same level in group S. Given these results, LF hypertrophy due to segmental hypermobility would have an important relationship with cartilage matrix increase. The ISC level in group F showed a significant decrease in the density of elastic fibres; thus, mechanical stress increase would lead to the disruption of elastic fibres and decrease of their density. Although the disruption of elastic fibres was actually observed in group F + C, the decrease in density with EVG stain did not reach significance. One of the possible reasons of the difference between groups F and F + C was from cartilage matrix increase on the ISC level in group F + C. The cartilage matrix was stained purple, whereas the elastic fibres was stained dark blue to black. Consequently, elastic fibres and cartilage matrix in the LF could not be separated from each other in image processing and significant difference in numerical value could not be detected.

Interestingly, slight disc height loss was identified in groups F and F + C, and had a weak but positive relationship with the number of *Col2a1* immunoreactive cells. Cartilage matrix increase in LF may be associated with disc degeneration. In a future study, we plan to develop a longer model and perform histological or gene analysis of the discs. In RT-PCR analysis, the *Col2a1* mRNA expression was upregulated in groups F and F + C on the ISC level compared with that on the same level in group S, whereas *Elastin* mRNA expression was significantly decreased. These findings support the results of our histological and immunohistological examinations. For fibrosis related gene, only *Col1a2* mRNA upregulation was observed in group F + C on the ISC level. Similar finding was reported by Yabe *et al*.^7^ in the tissue sample of patients with LSS. This suggests that fibrosis also has some relationship with LF hypertrophy following mechanical stress. However, we could not examine immunohistochemistry, because rabbit *Col1a2* primary antibody is not available.

Given these results, the increase in cartilage matrix and the decrease in elastic fibres density observed in this model are similar to the findings observed in thickened LF in patients with LSS. Therefore, this model should be useful for subsequent investigation on the biological mechanism of LF degeneration and hypertrophy obtained from intervertebral stress concentration^4,6^. The model of Group F + C, which performs cranio-caudal intervertebral fusion and supraspinal muscle resection, can obtain the increase in intervertebral range of motion in the early phase and indicate substantial chondrogenesis. Meanwhile, Group F is a suitable model to elucidate the correlation between LF degeneration and adjacent segment disease after lumbar spinal fusion, because we attempted to preserve posterior ligament complex during spinal fusion surgery for patients with LSS and never perform supraspinal ligament resection like group F + C.

Treatment of LSS has been used to perform decompression surgery to resect the LF with or without spinal fusion^11,17,18^. The paradigm shift of the last decade has been due to the introduction and widespread use of lateral lumbar interbody fusion (LLIF). We are able to manage with this system to fuse unstable levels without decompression^19^. The canal area of the patients who underwent LLIF without decompression were reported to be widen postoperatively compared with that preoperatively^20^. Some reports showed continuous expansion until 1 year after surgery^21^. However, the mechanism of the canal expansion and the effect of spinal fusion for the LF on fused levels are also unclear.

Based on the change in the LF on fused levels, we demonstrated in this study the decreased number of Col2a1 immunoreactive cells and the downregulation of not only *Col2a1* but also *Elastin* gene expression compared with the control. These results support the mechanism of LF shrinkage following lumbar fusion surgery in patients with LSS^20^. We failed to show the decrease in the density of elastic fibres histologically as opposed to *Elastin* gene downregulation on fused levels. This might be due to the short duration of the study period and inherently very little cartilage matrix in the normal LF.

This study has several limitations. First, the animal model was established using quadrupedal rabbits, therefore the spinal kinematics may be different from that of bipedal humans. In addition, the evaluation of intervertebral range of motion was manipulative. Whether a real-motion increase was obtained on the ISC level in fused models is unclear. Thus, this animal model may not represent the real degeneration process in human. However, it is difficult to use bipedal animals for the research purpose and create an animal model that replicates the degeneration in humans, which can take several decades. In this model, we can assume that the LF of ISC level had significant mechanical stress during the study periods postoperatively, and the histology showed significant changes similar with human degeneration. We believe that this model will further elucidate the mechanism of LF degeneration. Second, for the LF of ISC level in group F or F + C, the biochemical evaluation was not performed to determine why chondrogenesis occurred due to stress concentration. Inflammation is thought to contribute to LF degeneration and hypertrophy. Reports have demonstrated that proinflammatory cytokines have a crucial relationship with chondrogenesis^22,23^. In addition, we previously demonstrated that each BMP receptor type was higher in the LF obtained from patients with LSS and segmental hypermobility^4^. We are now performing microarray analysis of the LF, and studying the molecular mechanism of LF degeneration focusing on chondrogenesis and inflammatory cytokines, or BMPs. Third, for the pathological change in the LF on fused levels, this study revealed the findings of the fusion effect for the normal LF and spinal segment. Hence, we could not demonstrate the effect of fusion for degenerative spine and hypertrophied LF. The influence of spinal fusion for hypertrophied LF with subsequently upregulated *Col1a2*, *Col2a1* and proliferated cartilage matrix should be investigated in the future. However, we observed relevant findings that downregulation in various types of *Collagen* and *Elastin* would occur by spinal fusion for the LF. These results could help to explain why clinical and radiographical improvement would be obtained for patients with LSS by spinal fusion without decompression. Fourth, because black elastic fibre and purple cartilage matrix could not be separated from each other using binary data method with computer software, we could not measure exact distinction between the elastic fibres and cartilage matrix on ISC level in group F + C in EVG staining. However, even on ISC level in group F which less mechanical stress concentrated in motion segment compared to that of group F + C, the decrease of elastic fibres was observed. Furthermore, *Elastin* gene expression was downregulated on ISC level in group F + C compared to that of group S. We could presume mechanical stress concentration caused elastic fibres disruption.

In conclusion, we established a novel rabbit model that can evaluate the LF in LSS under standardised condition. Hypertrophy with increase in cartilage matrix and decrease in the density of elastic fibres was observed in the LF on ISC level compared with the same level of sham group. Col2a1 immunoreactive cells and gene expression were increased, whereas *Elastin* expression was decreased. On the other hand, not only Col2a1 immunoreactive cells and gene expression but also *Elastin* gene expression was decreased in the LF on fused levels compared with the same level of sham group.

## Electronic supplementary material

Supplementary File
