# Supplementary material for: Mechanical stress induces elastic fibre disruption and cartilage matrix increase in ligamentum flavum
Source: Sci Rep. 2017 Oct 12;7:13092. doi: 10.1038/s41598-017-13360-w (PMC5638934; doi:10.1038/s41598-017-13360-w)
Supplement: Supplementary file 1 — Supplementary File [file 41598_2017_13360_MOESM1_ESM.pptx]

## Slide 1
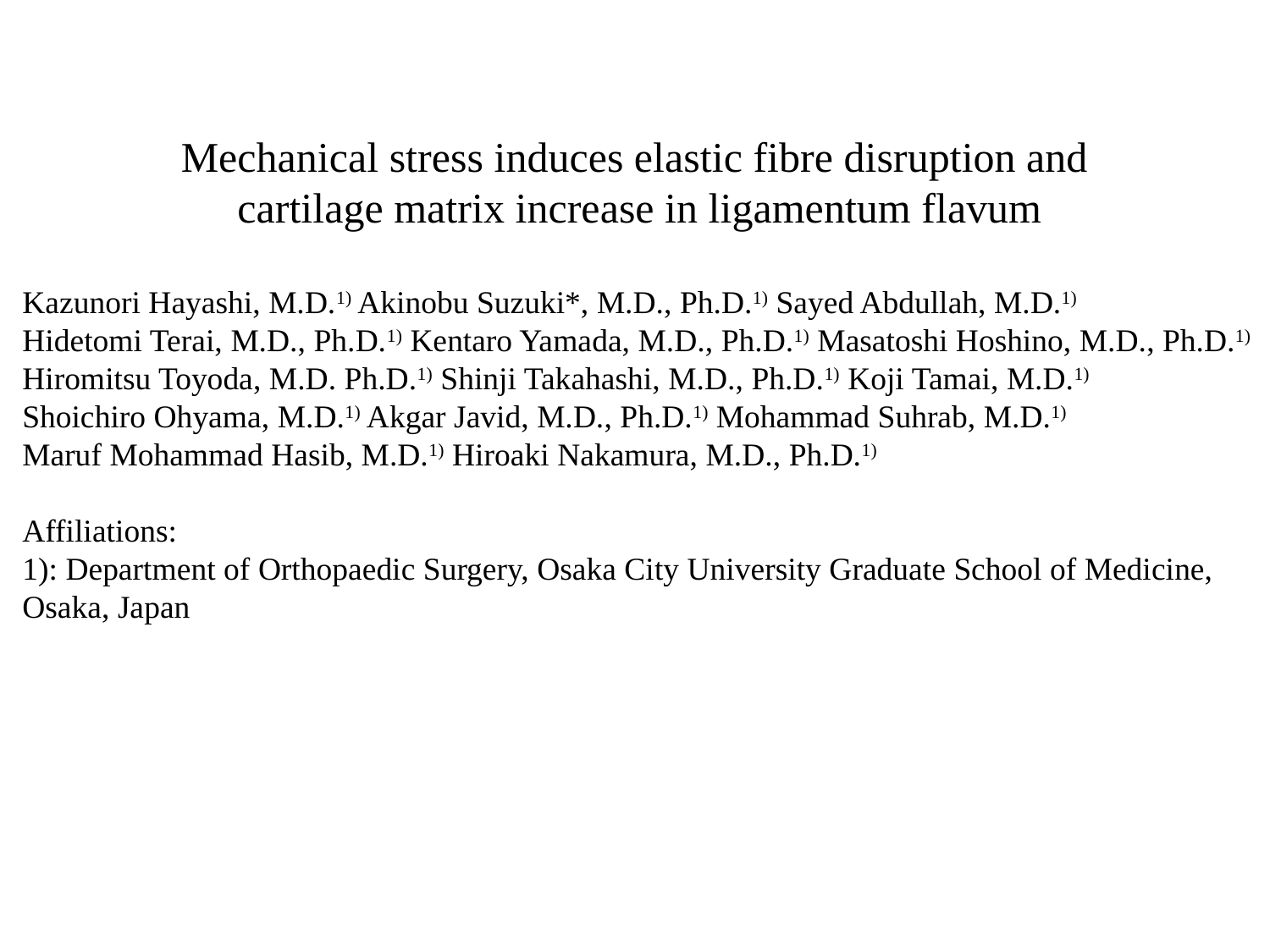

Mechanical stress induces elastic fibre disruption and
cartilage matrix increase in ligamentum flavum
Kazunori Hayashi, M.D.1) Akinobu Suzuki*, M.D., Ph.D.1) Sayed Abdullah, M.D.1)
Hidetomi Terai, M.D., Ph.D.1) Kentaro Yamada, M.D., Ph.D.1) Masatoshi Hoshino, M.D., Ph.D.1) Hiromitsu Toyoda, M.D. Ph.D.1) Shinji Takahashi, M.D., Ph.D.1) Koji Tamai, M.D.1)
Shoichiro Ohyama, M.D.1) Akgar Javid, M.D., Ph.D.1) Mohammad Suhrab, M.D.1)
Maruf Mohammad Hasib, M.D.1) Hiroaki Nakamura, M.D., Ph.D.1)
Affiliations:
1): Department of Orthopaedic Surgery, Osaka City University Graduate School of Medicine, Osaka, Japan

## Slide 2
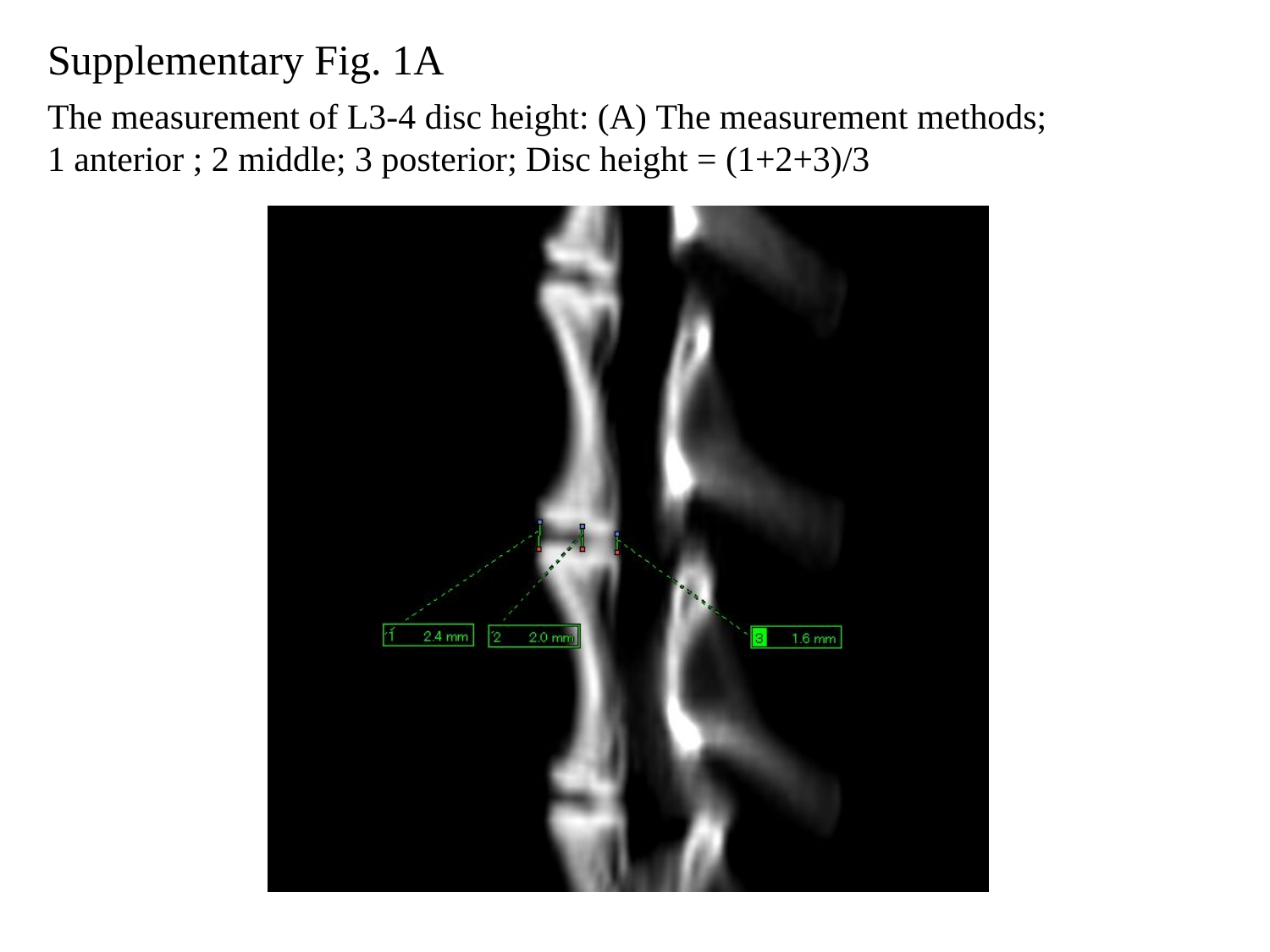

Supplementary Fig. 1A
The measurement of L3-4 disc height: (A) The measurement methods;
1 anterior ; 2 middle; 3 posterior; Disc height = (1+2+3)/3

## Slide 3
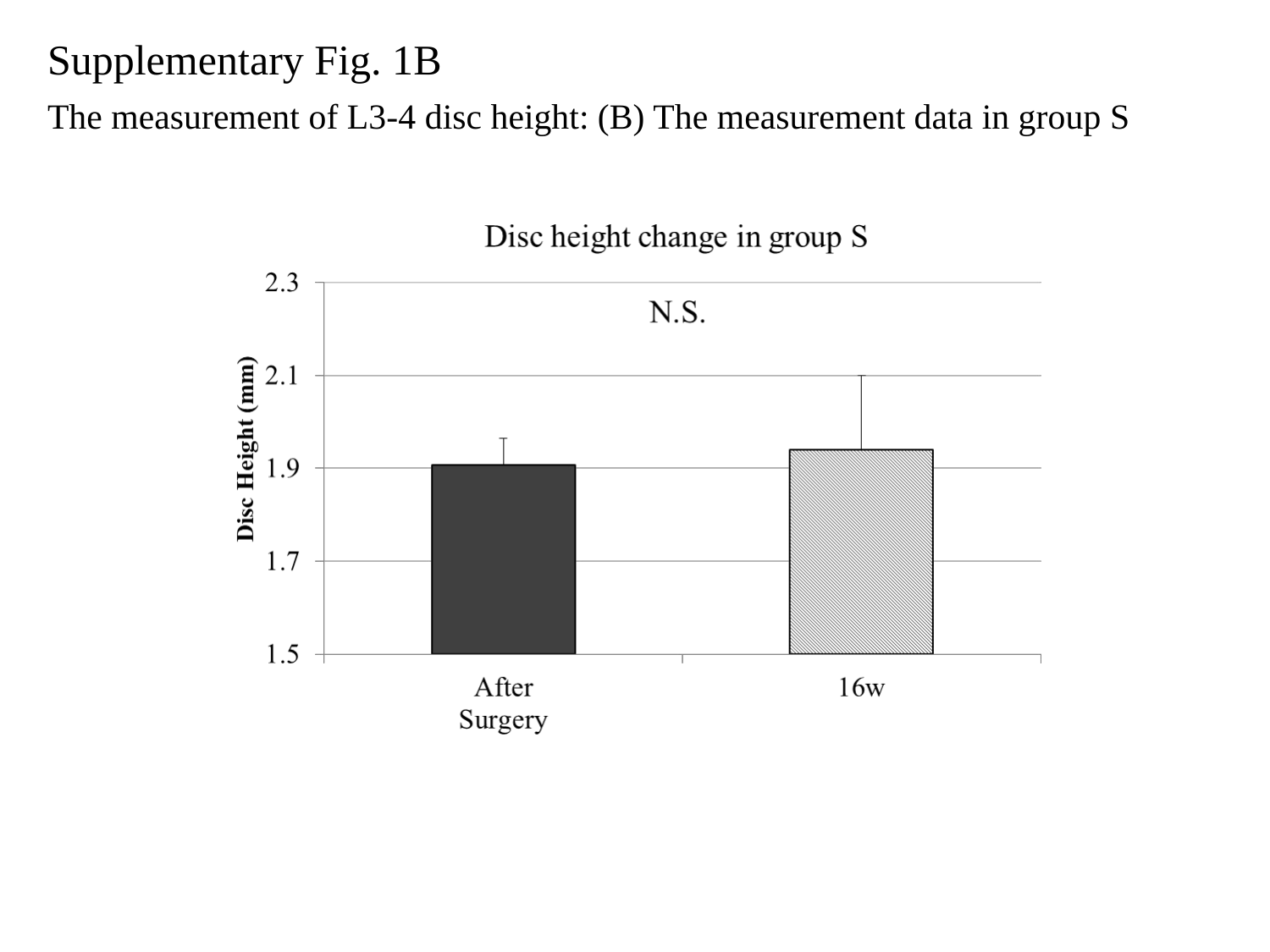

Supplementary Fig. 1B
The measurement of L3-4 disc height: (B) The measurement data in group S

## Slide 4
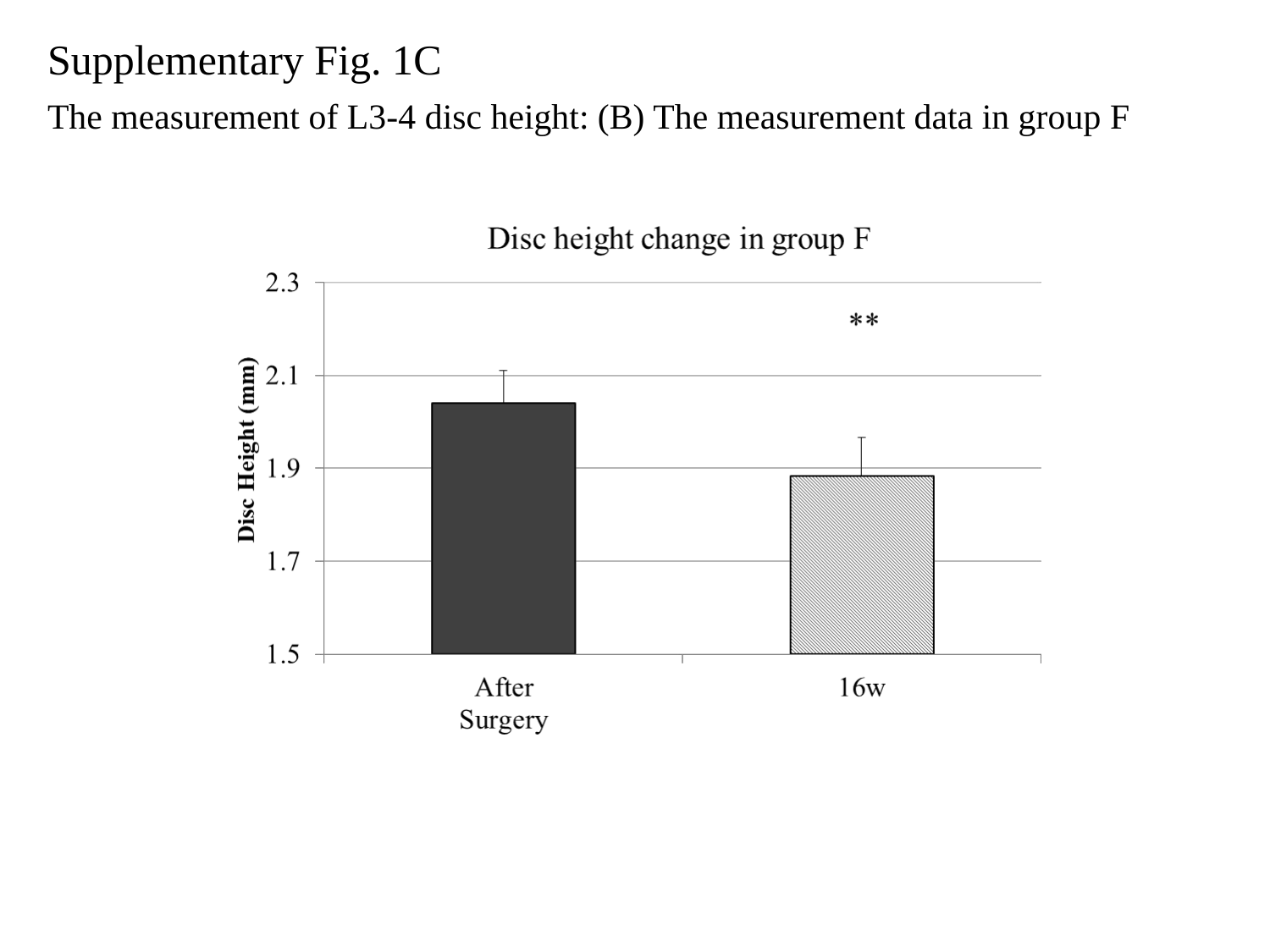

Supplementary Fig. 1C
The measurement of L3-4 disc height: (B) The measurement data in group F

## Slide 5
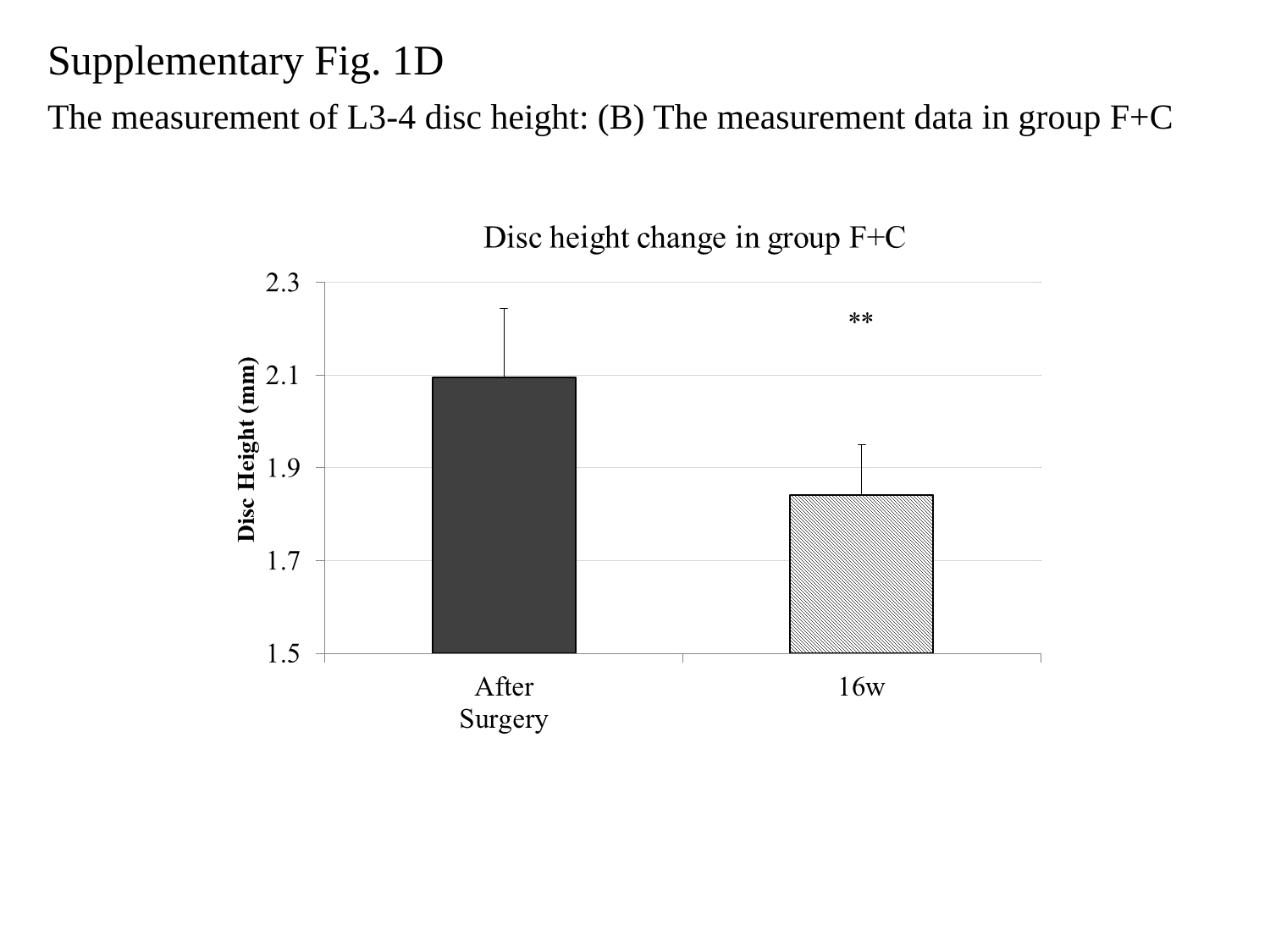

Supplementary Fig. 1D
The measurement of L3-4 disc height: (B) The measurement data in group F+C

## Slide 6
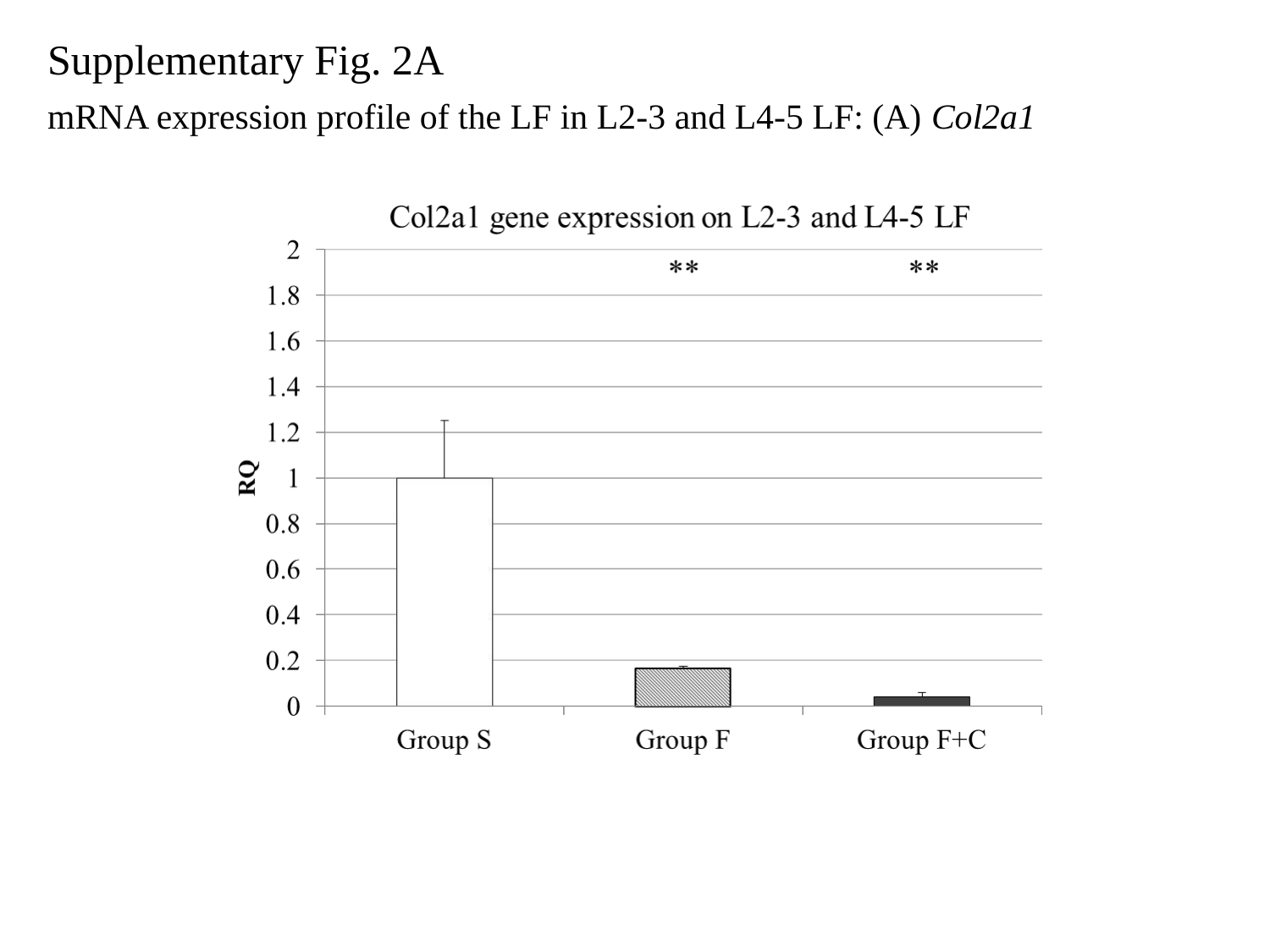

Supplementary Fig. 2A
mRNA expression profile of the LF in L2-3 and L4-5 LF: (A) Col2a1

## Slide 7
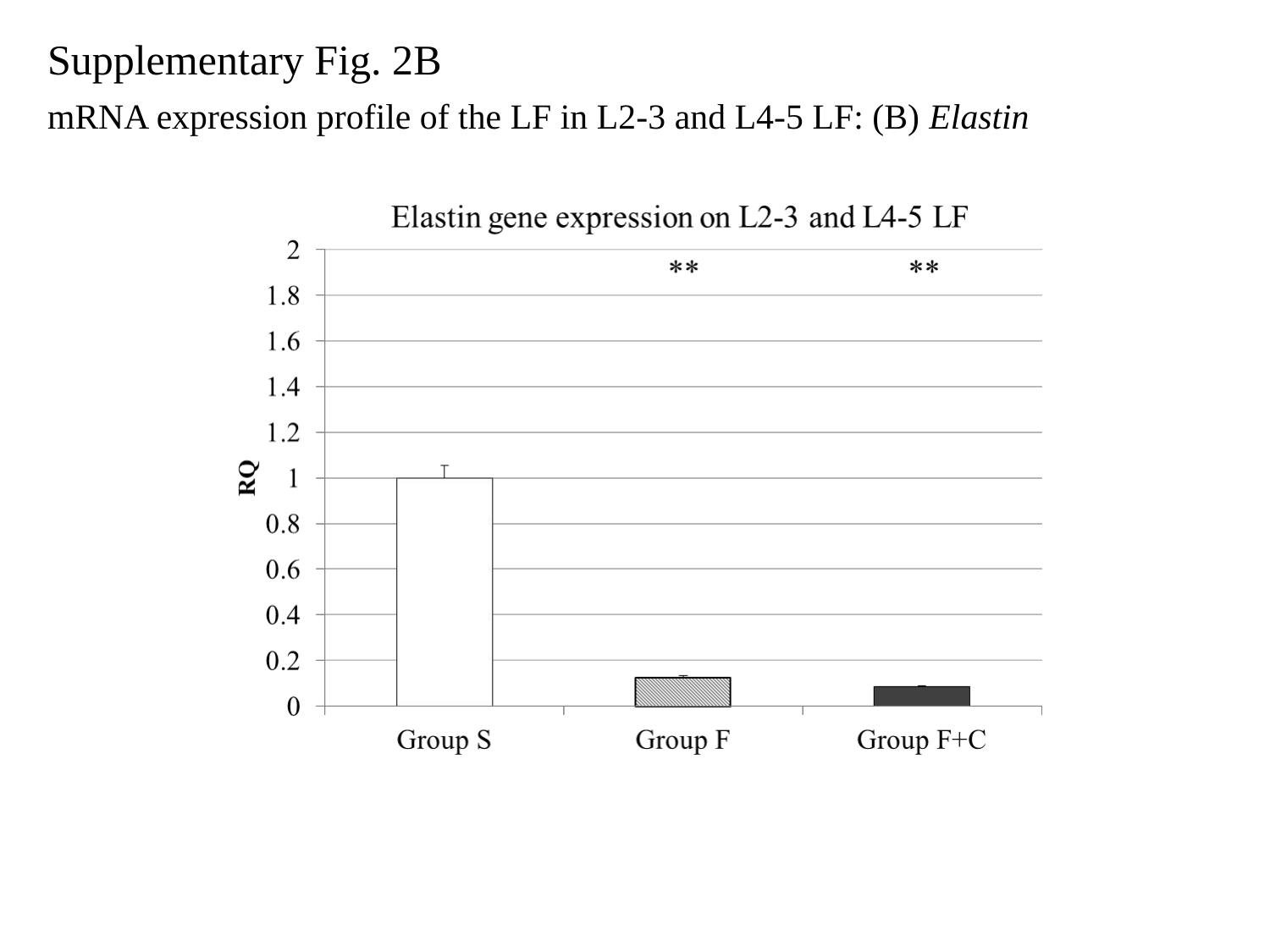

Supplementary Fig. 2B
mRNA expression profile of the LF in L2-3 and L4-5 LF: (B) Elastin

## Slide 8
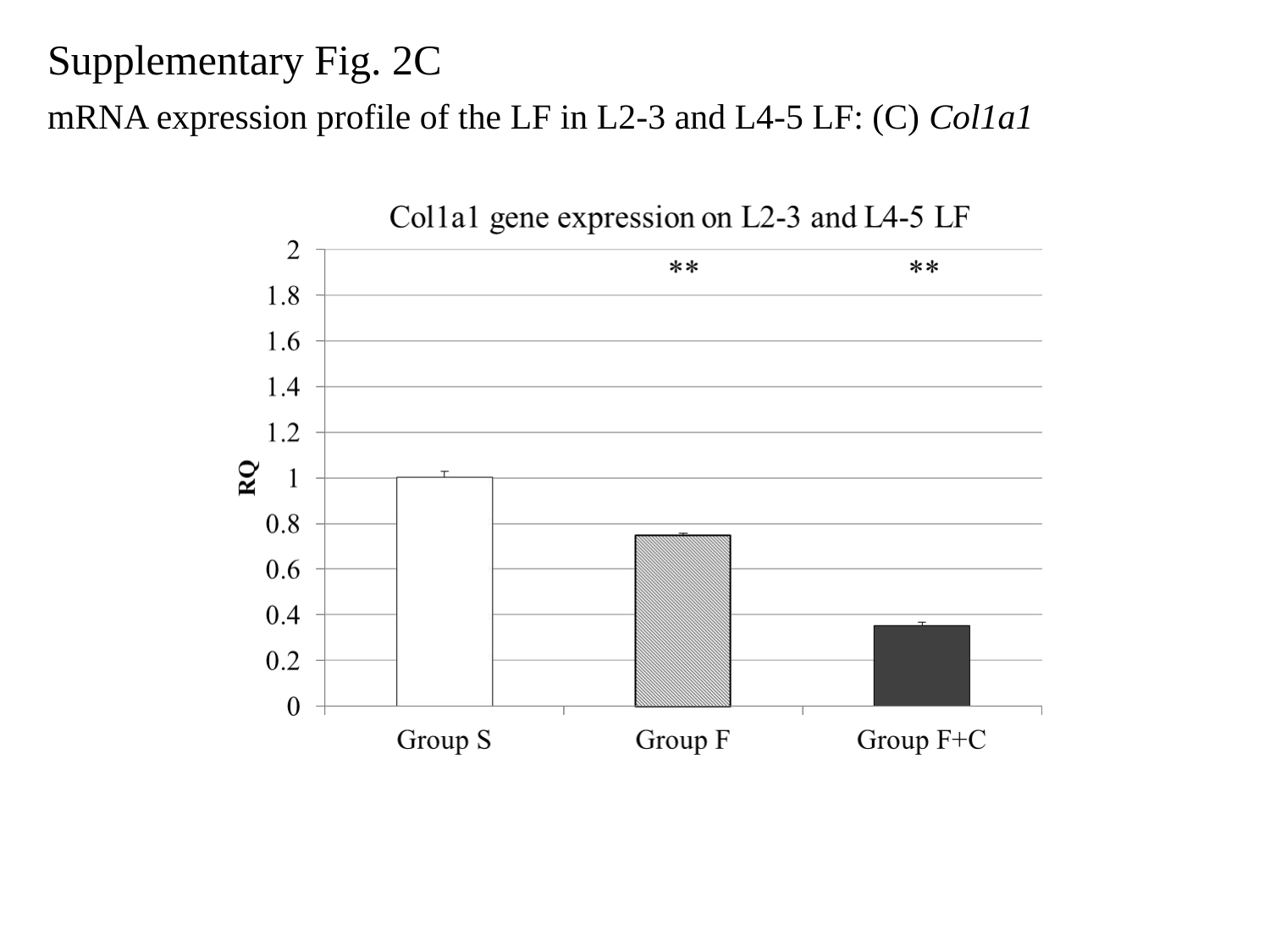

Supplementary Fig. 2C
mRNA expression profile of the LF in L2-3 and L4-5 LF: (C) Col1a1

## Slide 9
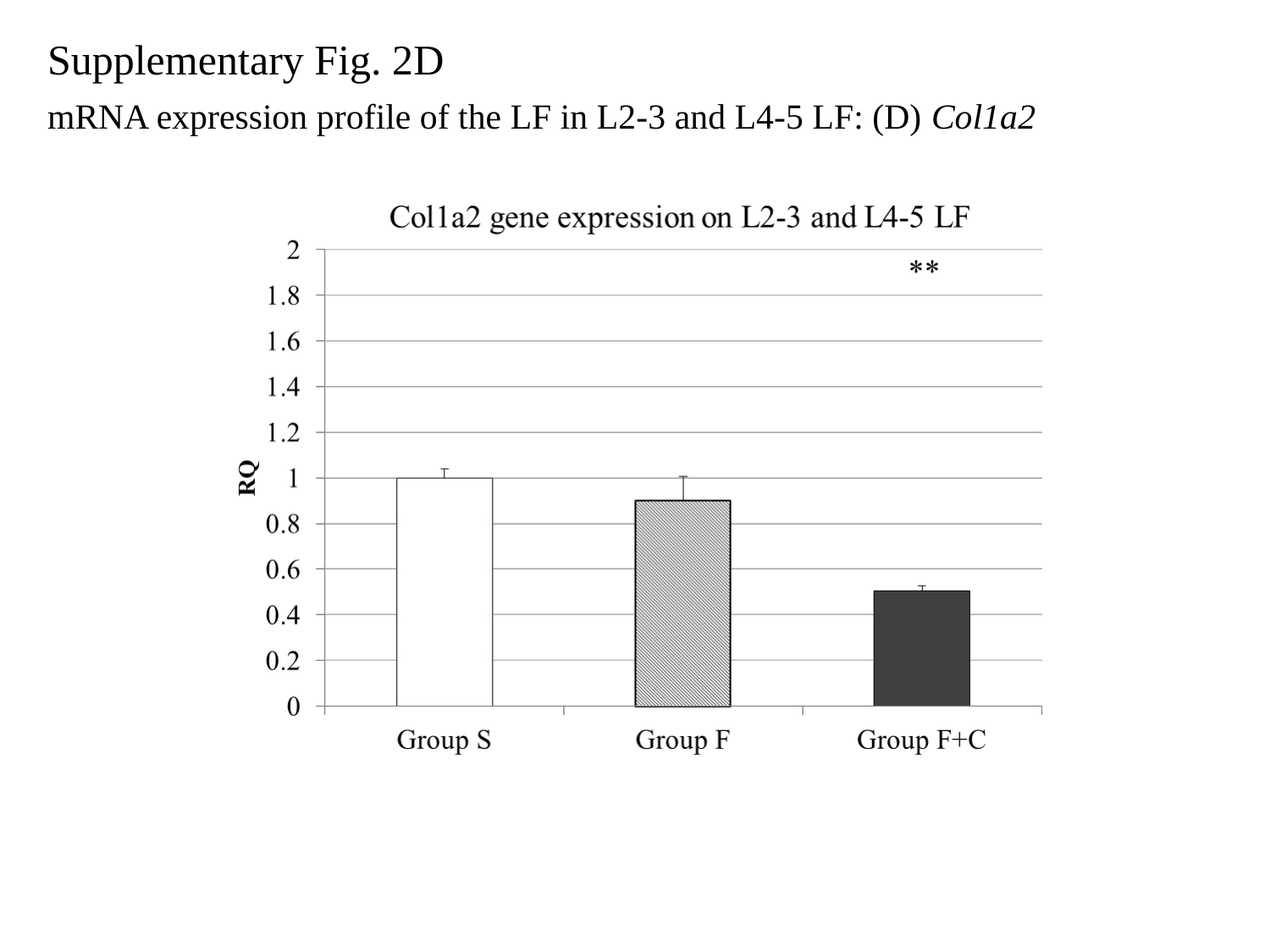

Supplementary Fig. 2D
mRNA expression profile of the LF in L2-3 and L4-5 LF: (D) Col1a2

## Slide 10
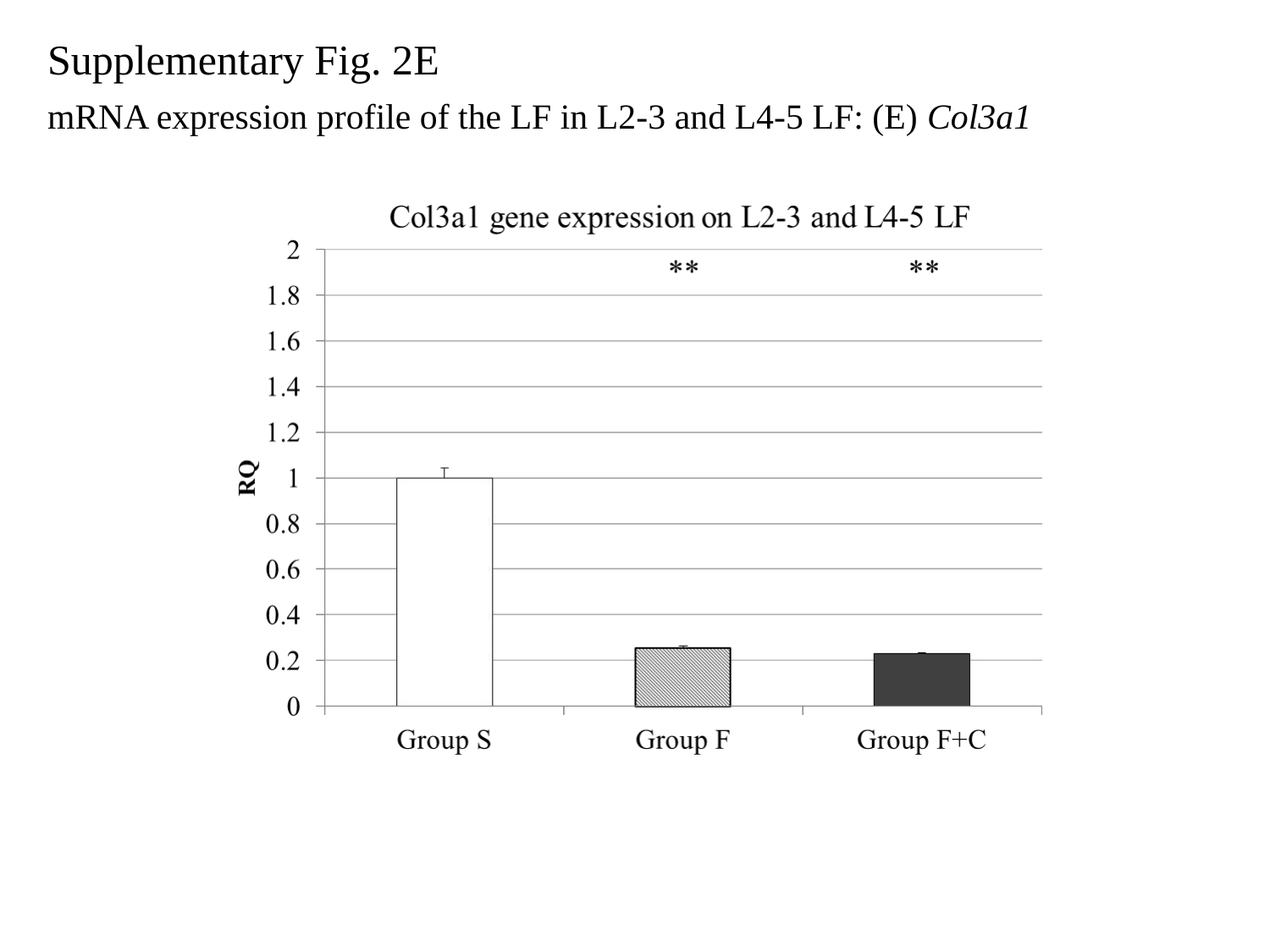

Supplementary Fig. 2E
mRNA expression profile of the LF in L2-3 and L4-5 LF: (E) Col3a1
